# Blood Tumor Mutational Burden as a Predictive Biomarker in Patients With Advanced Non-Small Cell Lung Cancer (NSCLC)

**DOI:** 10.3389/fonc.2021.640761

**Published:** 2021-05-14

**Authors:** Yuhui Ma, Quan Li, Yaxi Du, Jingjing Cai, Wanlin Chen, Guangqiang Zhao, Xing Liu, Hongsheng Li, Luyao Ma, Yunchao Huang, Yongchun Zhou

**Affiliations:** ^1^ First Department of Thoracic Surgery, The Yunnan Cancer Hospital, Kunming, China; ^2^ Department of Key Laboratory of Lung Cancer, Key Laboratory of Lung Cancer Research of Kunming Medical University, Kunming, China; ^3^ Yunnan Cancer Hospital and The Third Affiliated Hospital of Kunming Medical University, Yunnan Cancer Center, Kunming, China

**Keywords:** blood tumor mutational burden, next-generation sequencing, biomarker, immunotherapy, NSCLC

## Abstract

This study was designed to investigate the impact of blood tumor mutational burden (bTMB) on advanced NSCLC in Southwest China. The relationship between the tTMB estimated by next-generation sequencing (NGS) and clinical outcome was retrospectively analyzed in tissue specimens from 21 patients with advanced NSCLC. Furthermore, the relationship between the bTMB estimated by NGS and clinical outcome was retrospectively assessed in blood specimens from 70 patients with advanced NSCLC. Finally, 13 advanced NSCLC patients were used to evaluate the utility of bTMB assessed by NGS in differentiating patients who would benefit from immunotherapy. In the tTMB group, tTMB ≥ 10 mutations/Mb was related to inferior progression-free survival (PFS) (hazard ratio [HR], 0.30; 95% CI, 0.08-1.17; log-rank *P* = 0.03) and overall survival (OS) (HR, 0.30; 95% CI, 0.08-1.16; log-rank *P* = 0.03). In the bTMB group, bTMB ≥ 6 mutations/Mb was associated with inferior PFS (HR, 0.32; 95% CI, 0.14-1.35; log-rank *P* < 0.01) and OS (HR, 0.31; 95% CI, 0.14-0.7; log-rank *P* < 0.01). In the immunotherapy section, bTMB ≥ 6 mutations/Mb was related to superior PFS (HR, 0.32; 95% CI, 0.14-1.35; log-rank *P* < 0.01) and objective response rates (ORRs) (bTMB < 6: 14.2%; 95% CI, 0.03-1.19; bTMB ≥ 6: 83.3%; 95% CI, 0.91-37.08; *P* = 0.02). These findings suggest that bTMB is a validated predictive biomarker for determining the clinical outcome of advanced NSCLC patients and may serve as a feasible predictor of the clinical benefit of immunotherapies (anti-PD-1 antibody) in the advanced NSCLC population in Yunnan Province.

## Introduction

Recently, immune checkpoint blockade (ICB) therapy has shown improved clinical benefits in patients with advanced non-small cell lung cancer (NSCLC) (1, 2). Tissue with high tumor mutational burden (tTMB-H), which is related to genomic instability and overall neoantigen load, is a new prognostic biomarker for clinical benefit to multiple ICB therapies (3–5). More recent studies have demonstrated a relationship between tTMB-H and superior clinical benefit in patients receiving anti-PD-1 (anti cognate ligand of programmed death 1) antibodies therapy (6–10). However, up to 30% to 50% of patients with advanced NSCLC cannot supply sufficient cancer tissue for TMB detection (9, 11). Therefore, there is an urgent need to create noninvasive methods that can differentiate patients who would benefit from ICB therapy.

More recent studies have demonstrated that blood tumor mutational burden (bTMB, measured by circulating tumor DNA [ctDNA]) may be a substitution for overall neoantigen production (12–14). Gandara et al. (12) found an association between high bTMB (≥ 16 mutations/Mb) and superior progression-free survival (PFS) with atezolizumab in NSCLC. Zhijie Wang et al. (13) reported that bTMB (≥ 6 mutations/Mb) is related to PFS and that objective response rates (ORRs) benefit from ICB therapy in NSCLC. However, most previous studies have shown that there is not a relationship between high bTMB and greater overall survival (OS) in NSCLC patients received ICB therapy (12, 13, 15). Moreover, whether bTMB can be estimated by ctDNA is still unclear (16). Therefore, further evidence of bTMB is needed to identify its utility value as a biomarker for immunotherapy.

bTMB testing facilities (by NGS) have been established in our cancer center since 2018. The bTMB status in advanced NSCLC patients in the Yunnan Province is not clear, so we first investigated advanced NSCLC with bTMB in Yunnan Province and further determined the feasibility of bTMB as a prognostic biomarker for ICB therapy.

## Materials and Methods

### Patients

This research included 2 sections: we first recruited 21 advanced NSCLC patients (all of patients receiving platinum-based chemotherapy) and obtained tissue samples for retrospectively determining their tTMB status from the Yunnan Cancer hospital between February 2018 and August 2020. Second, we recruited 83 advanced NSCLC patients (70 patients receiving platinum-based chemotherapy and 13 patients receiving ICB therapy) with blood samples for retrospectively determining their bTMB status from the same center between January 2018 and August 2020. This work was performed in full accordance with the Declaration of Helsinki, and each patient provided informed consent. Protocol approval was acquired from the ethics committee of the Yunnan Cancer hospital. Advanced NSCLC patients from Yunnan Province with sufficient tissue or blood samples were considered eligible for this TMB analysis. Patients with known EGFR mutations or ALK translocations sensitive to targeted therapy or with an autoimmune disease were excluded. Twenty-one tissue samples underwent tTMB analysis, and 83 blood samples underwent bTMB analysis to obtain PFS and OS data. Finally, we collected other information, including clinical and molecular parameters.

### DNA Extraction and NGS Library Construction

Plasma ctDNA extraction and purification [Thermo (k0782)], leukocyte enrichment from whole blood, and low-speed centrifugation for DNA extraction and quality identification (Qubit dsDNA HS Assay Kit) were performed before library construction. The ctDNA libraries were prepared by the Vazyme ND607 DNA Library Kit with unique identifiers (UIDs, also called barcodes) to tag individual DNA molecules. The extracted DNA was used for end repair and poly(A)-tail addition by KAPA enzymatic digestion. Target fragments were captured by magnetic beads, and PCR enrichment amplification was performed after hybridization.

The tumor tissue DNA extraction and NGS library construction were performed following the methods described in our previous study (17).

### Sequencing and Data Processing

Captured probes were supplied by Integrated DNA Technologies (IDT) xGen Lockdown Probes (including 547 cancer-relevant genes) to evaluate the TMB, and then the probes were separately used to capture tissue gDNA and plasma ctDNA following standard protocols. Both captured libraries for tissue gDNA and plasma ctDNA were processed into the Illumina^®^ HiSeq X-TEN for sequencing according to the manufacturer’s instructions. Both raw parameters were processed by using Illumina^®^ HiSeq X-TEN, and error reads were corrected by using the hg19 reference genome. Our program KEYseq V2.0 was used to analyze these data.

### bTMB Detection

The TMB was calculated by summing all synonymous and nonsynonymous variants with ≥ 5% (0.5% in blood) frequency, which was demonstrated with the mutations per megabase (mut/Mb) unit, and germline variants and driver gene mutations were removed. Our bTMB algorithm contains single nucleotide polymorphism (SNP) sites and driver gene mutations filtering common untrue mutations, while synonymous mutations, small fragments and single base insertion-deletion mutations were not filtered. Our bTMB algorithm is based on the latest official standards of Foundation Medicine, a leading provider of genome sequencing analysis services. The tTMB cutoff point was 10 mutations/Mb, and the bTMB cutoff points were 6, 10, and 16 mutations/Mb based on recent studies (12, 13, 17).

### Outcomes Assessment

In the group of patients who did not receive ICB therapy, PFS was defined as the time from the start of TMB detection to objective disease progression or death, and this was evaluated with the Response Evaluation Criteria In Solid Tumors (RECIST) v 1.1 or death. OS was defined as the time from the start of TMB detection until death due to any cause. In the group of patients who received ICB therapy, PFS was defined as the time from the start of ICB treatment to objective disease progression or death. OS was defined as the time from the start of ICB treatment to death due to any cause. The ORR is measured as the percentage of patients with a complete response (CR) or a partial response (PR) (as defined by RECIST v1.1). Tumor assessments were performed every 3 months after the first TMB detection, and the last follow-up was on August 8, 2020.

### Statistical Analysis

Chi-square or Fisher’s exact test was used to analyze the correlation between TMB and clinical data. For survival analysis, Kaplan-Meier curves (*P* values determined with log-rank test) were performed, and the hazard ratios (HRs) were calculated by using a Cox proportional hazards regression model. *P* levels < 0.05 (two-sided) were considered statistically significant. All statistical analyses were performed by SPSS 22.0 (SPSS, Inc., Chicago, IL, USA) and Graph-Pad Prism version 8.0 (San Diego, CA, USA).

## Results

### Patient Characteristics

This study included 2 independent cohorts. Cohort 1 consisted of 21 patients with tissue samples; median age, 53 (range 36-75) years; 7 (33.3%) females. Cohort 2 consisted of 83 patients with blood samples; median age, 56 (range 31-82) years; 30 (36.1%) females. Thirteen patients received anti-PD-1 antibodies therapy as a second-line treatment. No evident differences were found in the patient characteristics (**eTables 1–3** in the **Supplement**).

### tTMB Estimated by NGS and Clinical Outcomes of NSCLC Patients

**Figure 1:**
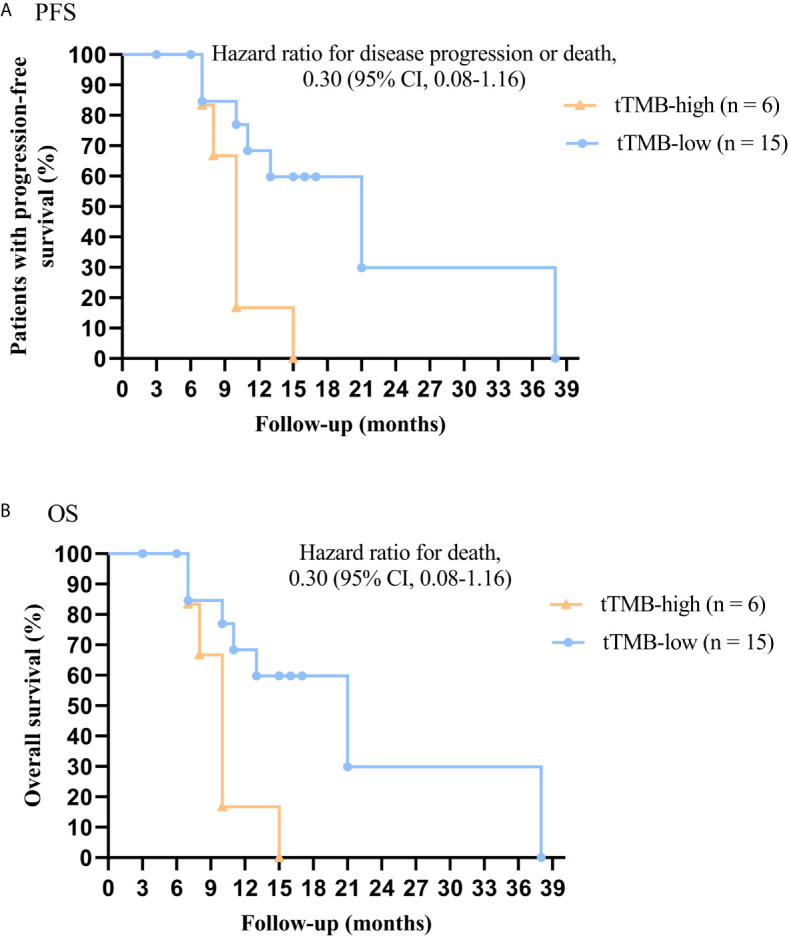
Kaplan-Meier plots of PFS and OS of patients by tTMB status: **(A)** PFS by tTMB status. **(B)** OS by tTMB status.

In cohort 1, the cutoff level of bTMB was 10 mutations/Mb based on previous studies and FDA guidelines (4, 9). PFS and OS were evidently shorter in patients with tTMB-high (≥ 10 mutations/Mb) than in patients with tTMB-low (< 10 mutations/Mb). The median PFS was 8.5 months and 19.0 months, respectively (hazard ratio = 0.30, 95% CI 0.08 to 1.17, *p* = 0.03, **Figure 1A**). The median OS was 10.0 months and 21.0 months, respectively (hazard ratio = 0.30, 95% CI 0.08 to 1.16, *p* = 0.03, **Figure 1B**). In the univariable Cox proportional hazards regression model, the bTMB status was related to PFS and OS (HR, 3.87; 95% CI, 1.14-13.12; *p* = 0.03) (HR, 3.92; 95% CI, 1.15-13.35; *p* = 0.02) (**Tables 1**, **2**). In the multivariable Cox proportional hazards regression model, the bTMB status was also associated with PFS and OS (HR, 4.20; 95% CI, 1.02-17.20; *p* = 0.04) (HR, 4.20; 95% CI, 1.01-17.49; *p* = 0.04) (**Tables 1**, **2**).

**Table 1:** Univariable and multivariable analysis of PFS.

	Progression-Free Survival			
	Univariable Analysis		Multivariable Analysis	
Characteristic	HR (95% CI)	*P*	HR (95% CI)	*P*
Age < 65 *vs* ≥ 65 y	0.94 (1.19-4.50)	*0.94*	NA	*NA*
Male *vs* female	1.56 (0.46-5.25)	*0.46*	NA	*NA*
Current or former *vs* never smoker	3.57 (0.98-12.91)	*0.05*	NA	*NA*
tTMB ≥ 10 *vs* < 10	3.87 (1.14-13.12)	*0.03a*	4.20 (1.02-17.20)	*0.04^a^*

a This p value indicates a statistically significant difference. NA, not applicable.

**Table 2:** Univariable and multivariable analysis of OS.

	Overall survival			
	Univariable Analysis		Multivariable Analysis	
Characteristic	HR (95% CI)	*P*	HR (95% CI)	*P*
Age < 65 *vs* ≥ 65 y	0.99 (0.21-4.65)	*0.99*	NA	*NA*
Male *vs* female	1.53 (0.45-5.15)	*0.48*	NA	*NA*
Current or former *vs* never smoker	3.19 (0.89-11.42)	*0.07*	NA	*NA*
tTMB ≥ 10 *vs* < 10	3.92 (1.15-13.35)	*0.02^a^*	4.20 (1.01-17.49)	*0.04^a^*

a This p value indicates a statistically significant difference. NA, not applicable.

### bTMB Estimated by NGS and Clinical Outcomes of NSCLC Patient Who Did Not Receive ICB Therapy

**Figure 2:**
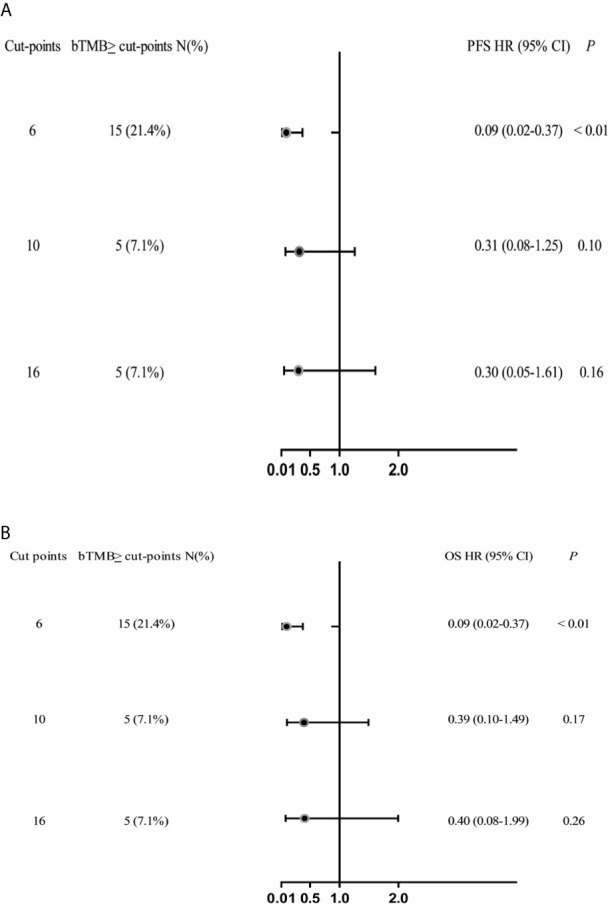
Forest plots of HRs for the relationship between bTMB cutoff values and PFS and OS. **(A)** PFS and **(B)** OS in the bTMB cohort (excluding patients who treated with ICB therapy), bTMB cutoff values of ≥6, ≥10 and ≥16.

**Figure 3:**
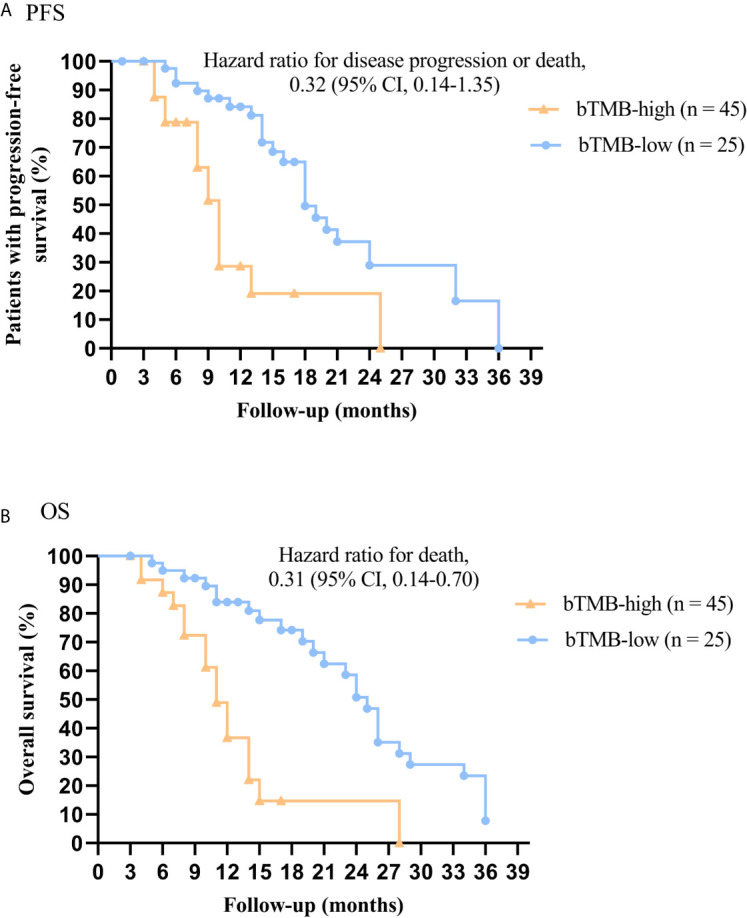
Kaplan-Meier plots of PFS and OS of patients (excluding patients who received ICB therapy) by bTMB status. **(A)** PFS by bTMB status. **(B)** OS by bTMB status.

In cohort 2, when the cutoff level of bTMB was 6, both HR and *P* levels were minimum values (**Figures 2A, B**). PFS and OS were evidently shorter in patients with bTMB-high (≥ 6 mutations/Mb) than in patient with bTMB-low (< 6 mutations/Mb). The median PFS was 10.0 months and 18.0 months, respectively (hazard ratio = 0.32, 95% CI 0.14 to 1.35, *p* < 0.01, **Figure 3A**). The median OS was 11.0 months and 25.0 months, respectively (hazard ratio = 0.31, 95% CI 0.14 to 0.7, *p* < 0.01, **Figure 3B**). In the univariable Cox proportional hazards regression model, bTMB status was related to PFS and OS (HR, 3.74; 95% CI, 1.85-7.54; *p* < 0.01) (HR, 4.48; 95% CI, 2.14-9.37; *p* < 0.01) (**Tables 3**, **4**). In the multivariable Cox proportional hazards regression model, bTMB status was also related to PFS and OS (HR, 4.20; 95% CI, 1.02-17.20; *p* = 0.04) (HR, 4.20; 95% CI, 1.01-17.49; *p* = 0.04) (**Tables 3**, **4**).

**Table 3:** Univariable and multivariable analysis of PFS.

	Progression-Free Survival			
	Univariable Analysis		Multivariable Analysis	
Characteristic	HR (95% CI)	*P*	HR (95% CI)	*P*
Age < 65 *vs* ≥ 65 y	1.62 (0.77-3.44)	*0.20*	NA	*NA*
Male *vs* female	1.67 (0.86-3.23)	*0.12*	NA	*NA*
Current or former *vs* never smoker	1.31 (0.70-2.46)	*0.39*	NA	*NA*
bTMB ≥ 6 *vs* < 6	3.74 (1.85-7.54)	*<0.01^a^*	5.35 (2.39-11.97)	*<0.01^a^*

a This p value indicates a statistically significant difference. NA, not applicable.

**Table 4:** Univariable and multivariable analysis of OS.

	Overall survival			
	Univariable Analysis		Multivariable Analysis	
Characteristic	HR (95% CI)	*P*	HR (95% CI)	*P*
Age < 65 *vs* ≥ 65 y	1.57 (0.74-3.31)	*0.23*	NA	*NA*
Male *vs* female	1.60 (0.85-3.00)	*0.14*	NA	*NA*
Current or former *vs* never smoker	1.46 (0.79-2.72)	*0.22*	NA	*NA*
bTMB ≥ 6 *vs* < 6	4.48 (2.14-9.37)	*<0.01^a^*	6.26 (2.71-14.46)	*<0.01^a^*

a This p value indicates a statistically significant difference. NA, not applicable.

### bTMB Estimated by NGS and Clinical Outcomes of NSCLC Patients Who Treated With ICB Therapy

**Figure 4:**
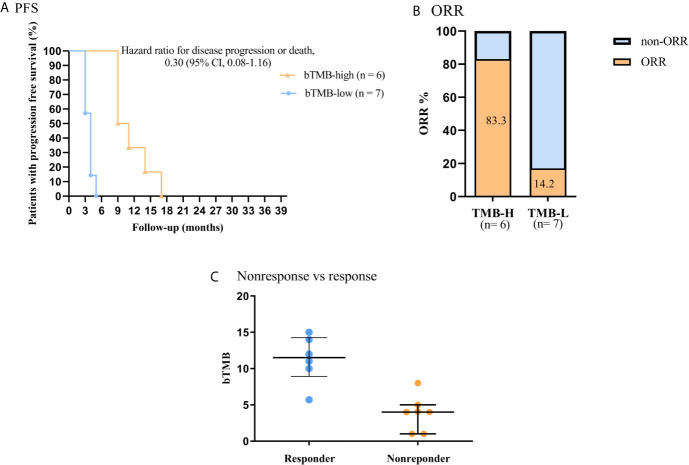
Association of clinical outcome and bTMB in NSCLC patients received ICB therapy. **(A)** PFS by bTMB status. **(B)** Comparison of ORRs in the two bTMB groups (*P* =0.02). **(C)** Comparison of bTMB values between the nonresponse and response groups (*P* < 0.01).

In this work, we further investigated the relationship between bTMB status and clinical benefit in advanced NSCLC patients who treated with ICB (anti-PD-1 antibody) therapy. The PFS was significantly shorter in patients with bTMB-low (< 6 mutations/Mb) than in patients with bTMB-high (< 6 mutations/Mb). The median PFS was 4.0 months and 10.0 months, respectively (hazard ratio = 3.96, 95% CI 1.083 to 14.48, *p* < 0.01, **Figure 4A**). Furthermore, bTMB-high was related to a higher ORR than bTMB-low (83.3%; 95% CI, 0.91-37.08 *vs*. 14.2%; 95% CI, 0.03-1.19; *P* = 0.02, **Figure 4B**). Eventually, nonresponders had significantly lower bTMB levels than responders (median, 4; interquartile range, 1-8 *vs*. median, 11.5; interquartile range, 5.7-15, *P* < 0.01, **Figure 4C**).

### Prevalence of Genetic Alterations in Patients

**Figure 5:**
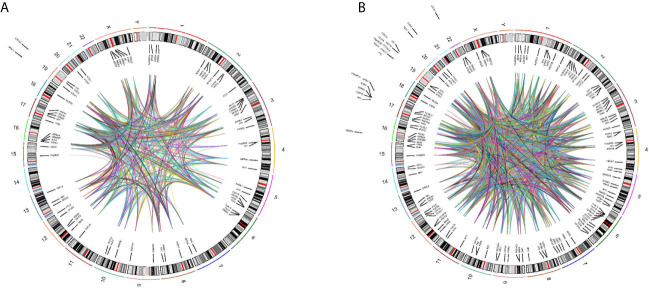
Circos plot describing whole-genome DNA-seq data. *Track 1*: cytoband, chromosomes are depicted qter to pter. *Track 2*: Genomic location of mutant genes in tissues **(A)** or blood **(B)** of detected patients is located inside or outside cytoband of related genome. Gene name is in black represent. *Track 3*: Each line represent mutant genes existing in a patient.

We found a wide range of cancer-related genetic alterations depended on tissue NGS genotyping. The landscape of genetic alterations had been shown in **Figure 5A**. The most frequently mutated genes were *TP53* (8 of 21, 38.1%) and *KRAS* (4 of 21, 19.0%). In addition, through plasma NGS genotyping, we also identified a wide range of cancer-related genetic alterations. The landscape of genetic alterations had been shown in **Figure 5B**. The most frequently mutated genes were *TP53* (29 of 83, 34.9%), *KRAS* (16 of 83, 19.3%) and *PIK3A* (12 of 83, 14.5%).

### Mutation Variant Allele Frequency (VAF) Distribution of All Patients

In the tissue VAF subgroup, we found that the median TP53 VAF was 35.77% (range 5.06% to 42.20%) in patients with tTMB-low, while it was 28.93% (range 13.12% to 57.85%) in patients with tTMB-high. The median TP53 VAF was 14.50% (5.06% to 57.85%) in patients with adenocarcinoma and 38.27% (21.33% to 51.70%) in patients with squamous cell carcinoma. However, there were no significant differences in the tumor type and TMB level (**eTable 4**). In the blood VAF subgroup, we found that the median TP53 VAF was 7.76% (range 1.62% to 73.16%) in patients with bTMB-low and 17.18% (range 0.97% to 79.10%) in patients with bTMB-high. The median TP53 VAF was 12.18% (1.27% to 79.10%) in patients with adenocarcinoma and 11.92% (4.24% to 62.12%) in patients with squamous cell carcinoma. The median KRAS VAF was 13.13% (range 2.57% to 59.07%) in patients with bTMB-low and 28.71% (range 5.05% to 61.21%) in patients with bTMB-high. Similarly, there were no significant differences in the tumor type and TMB level (**eTable 5**).

## Discussion

In this work, we first examined the association between the tTMB values and advanced NSCLC patients’ clinical outcomes who did not receive ICB therapy in Southwest China. Our findings were consistent with our previous study (17), and tTMB (cutoff value = 10 mutations/Mb) is a vital and independent predictive biomarker in advanced NSCLC. Many recent studies demonstrated that 30% to 50% of advanced NSCLC patients could not provide sufficient cancer tissue for tTMB detection (9, 12, 13). Therefore, whether bTMB could be a reliable biomarker to guide immunotherapies has attracted extensive attention from scientists. However, most of the research has focused on the relationship between bTMB values and advanced NSCLC patients’ clinical benefits from ICB therapy. The relationship between bTMB values and clinical outcomes in patients who did not receive ICB therapy is still not clear. Herein, this is the study that demonstrates that advanced NSCLC patients (receiving platinum-based chemotherapy) with bTMB-low (cutoff value < 6 mutations/Mb) were associated with better outcomes in Yunnan Province (**Figures 3A, B**). These findings are similar to our previous tTMB study (17). It may be inferred that bTMB values are important for estimating advanced NSCLC patients’ clinical outcomes and are similar to the outcomes estimated by tTMB levels.

This study also demonstrated that advanced NSCLC patients with bTMB-H (≥ 6 mutations/Mb) would benefit from ICB (anti-PD-1 antibody) therapy. These findings are also similar to those from previous studies (13). However, we also found that unlike tTMB, the OS benefit did not occur in advanced NSCLC patients with bTMB-H during ICB (anti-PD-1 antibody) therapy (1, 2). Therefore, more studies on bTMB are needed to investigate its intrinsic nature and reveal the potential mechanism.

The selection of bTMB cutoff points is still controversial (12, 13). Gandara et al. (12) and the phase III IMpower 110 study (18, 19) reported that advanced NSCLC patients with bTMB-H (≥ 16 mutations/Mb) would benefit from ICB therapy. More recently, MYSTIC study reported that advanced NSCLC patients with bTMB cutoff points (≥ 20 mutations/Mb) would benefit from ICB therapy (20). Subsequently, Zhijie Wang et al. (13) reported that advanced NSCLC patients with bTMB-H (≥ 6 mutations/Mb) would benefit from ICB therapy. In our study, we confirmed that the cutoff level of bTMB was 6 mutations/Mb, which is a suitable cutoff point that can differentiate advanced NSCLC patients from Yunnan Province who would benefit from ICB (anti-PD-1 antibody) therapy. The difference in the bTMB cutoff point between our findings and the other previous studies may result from five factors (12, 13, 18–20). First, the difference in the gene panel size between our research (547 cancer-relevant genes) and the study by Gandara et al. (394 cancer-relevant genes) may contribute to a discrepancy in the selection of the bTMB cutoff point. Second, the difference in patients’ race between our research (all patients were Chinese) and Gandara et al.’s study (most patients were White) may contribute to a bias in the selection of the bTMB cutoff point. Third, the limited sample size in our study (only 13 patients received anti-PD-1 antibody therapy) may lead to a bias in the selection of the bTMB cutoff point. Fourth, the difference in the calculation of bTMB between our research (summing all synonymous and nonsynonymous variants) and other studies (summing all nonsynonymous variants) may contribute to a bias in the selection of the bTMB cutoff point (20, 21). Finally, our study was a retrospective analysis that may contribute to a statistical discrepancy. Therefore, further prospective studies with large sample sizes are needed.

In our study, a comprehensive analysis of genetic alterations in NSCLC patients demonstrated that the majority of mutations appeared in TP53 and KRAS (**Figure 5**). These findings are similar to our previous study (17).

In summary, our findings suggest that bTMB (cutoff point was 6 mutations per megabase) is a validated predictive biomarker for determining the clinical outcome of advanced NSCLC patients with chemotherapy. Using a bTMB cutoff point ≥ 6 mutations per megabase, we found that Yunnan advanced NSCLC patients who obtained an increased PFS benefit from anti-PD-1 antibody therapy. Further prospectively validated studies with large sample sizes are needed.

There are three limitations to our study. Firstly, our study was retrospective research, which may limit the interpretation of the clinical results. Secondly, the small sample size may cause unavoidable selection bias and measurement bias, relatively weakening the reliability of our conclusions. Thirdly, in our study, there were no matched blood and clinical tissue samples from the same patient for TMB detection. Therefore, it may lead to a bias between tTMB and bTMB in diagnostic concordance assessment. Further prospective researches are expected.

## Data Availability Statement

The datasets presented in this study can be found in online repositories. The names of the repository/repositories and accession number(s) can be found in the article/**Supplementary Material**.

## Author Contributions

YZ, YH, and YM designed the study. YM, QL, YD, JC, WC, GZ, XL, HL, and LM finished the experiments. YM and YZ analyzed the parameters and wrote the research. All authors contributed to the research. All authors contributed to the article and approved the submitted version.

## Funding

This work was supported by the National Natural Science Foundation of China (No. 81860513).

## Conflict of Interest

The authors declare that the research was conducted in the absence of any commercial or financial relationships that could be construed as a potential conflict of interest.
