# Supplementary material for: Blood Tumor Mutational Burden as a Predictive Biomarker in Patients With Advanced Non-Small Cell Lung Cancer (NSCLC)
Source: Front Oncol. 2021 May 14;11:640761. doi: 10.3389/fonc.2021.640761 (PMC8160368; doi:10.3389/fonc.2021.640761)
Supplement: Supplementary file 1 [file Table_1.doc]

**eTable 1. Detailed Clinicopathologic Features of 21 NSCLC Patients for tTMB test.**

| |  | **Total** | **tTMB < 10** | **tTMB > 10** | ***p*** | | --- | --- | --- | --- | --- | | **Characteristic** |  | **(N = 15)** | **(N = 6)** |  | |
| --- | --- | --- | --- | --- | --- | --- | --- | --- | --- | --- |
| | No. of patients, N (%) | 21 | 15 (71.4) | 6 (28.6) |  | | --- | --- | --- | --- | --- | | **Age at initiation of TMB test**: Median (range), y | 53 (36-75) | 53 (36-71) | 56 (46-75) |  | | **TMB (mutations/Mb), median (range)** | 6 (0.6-27) | 4.1 (0.6-8) | 19.5 (11-27) |  | | **Sex, N (%)** |  |  |  |  | | Male | 14 (66.7) | 11 (78.6) | 3 (21.4) | 0.35 | | Female | 7 (33.3) | 4 (57.1) | 3 (42.9) |  | | **Histology, N (%)** |  |  |  |  | | Adenocarcinoma | 16 (76.2) | 12 (75.0) | 4 (25.0) | 0.59 | | Squamous cell carcinoma | 5 (23.8) | 3 (60.0) | 2 (40.0) |  | | **Smoking history, N (%)** |  |  |  |  | | Never | 13 (61.9) | 9 (69.2) | 4 (30.8) | 1 | | Former/current | 8 (38.1) | 6 (75.0) | 2 (25.0) |  | | **Tumor type, N (%)** |  |  |  |  | | Primary tumor | 20 (95.2) | 14 (70.0) | 6 (30.0) | 1 | | Metastasis / Lymph node | 1 (4.8) | 1 (100.0) | 0 (0.0) |  | | **Chemotherapy, N (%)** |  |  |  |  | | Yes | 21 (100.0) | 15 (71.4) | 6 (28.6) |  | | No | 0 (0.0) | 0 (0.0) | 0 (0.0) |  | |

**eTable 2. Detailed Clinicopathologic Features of 70 NSCLC Patients without ICBs for bTMB test.**

| | **Total** | **bTMB < 6** | **bTMB > 6** | ***p*** |  | | --- | --- | --- | --- | --- | | **Characteristic** |  | **(N = 45)** | **(N = 25)** |  | |
| --- | --- | --- | --- | --- | --- | --- | --- | --- | --- | --- |
| | No. of patients, N (%) | 70 | 45 (64.3) | 25 (35.7) |  | | --- | --- | --- | --- | --- | | **Age at initiation of TMB test**: Median (range), y | 56 (31-82) | 55 (31-82) | 59 (36-80) |  | | **TMB (mutations/Mb), median (range)** | 4.0 (0-49) | 3.0 (0-5.0) | 9 (6-49) |  | | **Sex, N (%)** |  |  |  |  | | Male | 43 (61.4) | 25 (58.1) | 18 (41.9) | 0.17 | | Female | 27 (38.6) | 20 (74.1)) | 7 (25.9) |  | | **Histology, N (%)** |  |  |  |  | | Adenocarcinoma | 59 (84.3) | 40 (67.8) | 19 (32.2) | 0.28 | | Squamous cell carcinoma | 11 (15.7) | 5 (45.5) | 6 (54.5) |  | | **Smoking history, N (%)** |  |  |  |  | | Never | 39 (55.7) | 27 (69.2) | 12 (30.8) | 0.33 | | Former/current | 31 (44.3) | 18 (58.1) | 13 (41.9) |  | | **Tumor type, N (%)** |  |  |  |  | | Primary tumor | 61 (87.1) | 40 (65.6) | 21 (34.4) | 0.83 | | Metastasis / Lymph node | 9 (12.9) | 5 (55.6) | 4 (44.4) |  | | **Chemotherapy, N (%)** |  |  |  |  | | Yes | 70 (100.0) | 45 (64.3) | 25 (35.7) | 0.59 | | No | 0 (0.0) | 0 (0.0) | 0 (0.0) |  | |

^a^ This *p* value indicates a statistically significant difference.

**eTable 3. Detailed Clinicopathologic Features of 13 NSCLC Patients with ICBs for bTMB test.**

| | **Total** | **bTMB < 6** | **bTMB > 6** | ***P*** |  | | --- | --- | --- | --- | --- | | **Characteristic** |  | **(N = 7)** | **(N = 6)** |  | |
| --- | --- | --- | --- | --- | --- | --- | --- | --- | --- | --- |
| | Characteristic |  | (N = 7) | (N = 6) |  | | --- | --- | --- | --- | --- | | No. of patients, N (%) | 13 | 7 (53.8) | 6 (46.2) |  | | **Age at initiation of TMB test**: Median (range), y | 56 (49-69) | 57 (50-65) | 54 (49-69) |  | | **TMB (mutations/Mb), median (range)** | 5.7 (1-15) | 4.0 (1-5.7) | 11.5 (8-15) |  | | **Sex, N (%)** |  |  |  |  | | Male | 10 (76.9) | 5 (50.0) | 5 (50.0) | 1 | | Female | 3 (23.1) | 2 (66.7) | 1 (33.3) |  | | **Histology, N (%)** |  |  |  |  | | Adenocarcinoma | 8 (61.5) | 5 (62.5) | 3 (37.5) | 0.59 | | Squamous cell carcinoma | 5 (38.5) | 2 (40.0) | 3 (60.0) |  | | **Smoking history, N (%)** |  |  |  |  | | Never | 6 (46.2) | 4 (66.7) | 2 (33.3) | 0.59 | | Former/current | 7 (53.8) | 3 (42.9) | 4 (57.1) |  | | **Tumor type, N (%)** |  |  |  |  | | Primary tumor | 9 (69.2) | 4 (44.4) | 5 (55.6) | 0.55 | | Metastasis / Lymph node | 4 (30.8) | 3 (75.0) | 1 (25.0) |  | | **Immunotherapy (anti-PD-1 antibodies)** |  |  |  |  | | Nivolumab | 4 (4.8) | 3 (75.0) | 1 (25.0) | 0.55 | | Pembrolizumab | 9 (10.8) | 4 (44.4) | 5 (55.6) |  | |

^a^ This *p* value indicates a statistically significant difference.

**eTable 4. Comparison of variant allele frequencies in tTMB level and tumor type.**

| | VAF (Median, Range) | tTMB < 10 (N = 15) | tTMB > 10 (N = 6) | *P* | LUAD (N = 16) | LUSC (N = 5) | *P* | | --- | --- | --- | --- | --- | --- | --- | |
| --- | --- | --- | --- | --- | --- | --- | --- |
| | TP53 | 35.77% (5.06%-42.20%) | 28.93% (13.12%-57.85%) | 0.31 | 14.50% (5.06%-57.85%) | 38.27% (21.33%-51.70%) | 0.14 | | --- | --- | --- | --- | --- | --- | --- | | KRAS | NA | NA | NA | 15.90% (6.39%-25.4%) | NA | NA | |

^a^ This *p* value indicates a statistically significant difference. LUAD, Lung adenocarcinoma. LUSC, Lung squamous cell carcinoma. NA, not applicable. VAF, variant allele frequency.

**eTable 5. Comparison of variant allele frequencies in bTMB level and tumor type.**

| | VAF (Median, Range) | bTMB < 6 (N) | bTMB > 6 (N) | *P* | LUAD (N = 67) | LUSC (N = 16) | *P* | | --- | --- | --- | --- | --- | --- | --- | |
| --- | --- | --- | --- | --- | --- | --- | --- |
| | TP53 | 7.76% (1.62%-73.16%) | 17.18% (0.97%-79.10%) | 0.07 | 12.18% (1.27%-79.10%) | 11.92%（4.24%-62.12） | 0.38 | | --- | --- | --- | --- | --- | --- | --- | | KRAS | 13.13% (2.57%-59.07%) | 28.71% (5.05%-61.21%) | 0.44 | 27.40%（2.57%-61.21%） | NA | NA | | PIK3CA | 19.31% (1.72%-36.90%) | 5.03% (0.66%-34%) | NA | 2.13% (0.66%-34%) | 22.31% (7.72%-36.9%) | NA | |

^a^ This *p* value indicates a statistically significant difference. LUAD, Lung adenocarcinoma. LUSC, Lung squamous cell carcinoma. NA, not applicable. VAF, variant allele frequency.
